# Ep-CAM (MOC-31) expression in tooth germ and ameloblastoma

**DOI:** 10.4317/medoral.25145

**Published:** 2022-08-17

**Authors:** Nathalie Derderian, Vanesa Pereira-Prado, Marcela Hernandez, Mario Isiordia-Espinoza, Miguel Arocena, Rogelio González-González, Omar Tremillo-Maldonado, Marco Meleti, Nelly Molina-Frechero, Ronell Bologna-Molina

**Affiliations:** 1 Molecular Pathology, School of Dentistry Universidad de la República (UDELAR), Montevideo, Uruguay; 2 Periodontal Biology Laboratory, Faculty of Dentistry and Department of Oral Pathology and Medicine Faculty of Dentistry University of Chile. University of Chile. Santiago, Chile; 3 Instituto de Investigación en Ciencias Médicas, Departamento de Clínicas, División de Ciencias Biomédicas, Centro Universitario de los Altos, Universidad de Guadalajara, Tepatitlán de Morelos, Mexico; 4 Biochemistry and Biophysics, School of Dentistry Universidad de la República (UDELAR), Montevideo Uruguay; 5 Department of Research, School of Dentistry, Universidad Juárez del Estado de Durango, Durango, Mexico; 6 Centro Universitario di Odontoiatria, Department of Medicine and Surgery, University of Parma, Parma, Italy; 7 Departamento de Atención a la Salud, Universidad Autónoma Metropolitana (UAM) Unidad Xochimilco, Ciudad de México, Mexico

## Abstract

**Background:**

Ep-CAM, a transmembrane glycoprotein expressed in most epithelium in normal conditions, has diverse roles in these tissues, including in cell adhesion, proliferation, differentiation, cell cycle regulation, migration and intracellular signaling. It is also over-expressed in most malignant neoplasia, participating in the initiation, progression, and metastatic dissemination of the tumor. The expression and roles of this protein in oral neoplasia, particularly in odontogenic tumors, remain unestablished. The objective of this study consisted in analyzing the expression of this protein in ameloblastoma and tooth germ.

**Material and Methods:**

Ep-CAM (MOC-31) expression was evaluated by immunohistochemistry in tooth germs (TG) (n = 16) ameloblastomas (AM) (n = 60) and 2 ameloblastic carcinomas. Sections were visualized in their totality with an optical microscope, and positivity observed in cell membrane and cytoplasm was graded according to the following semi-quantitative scale: Neg, "essentially unstained", for negative sections or staining <5% of cells; + for staining of 5-50% of cells; ++ for staining >50% of cells.

**Results:**

Most tooth germs expressed MOC-31 (81.3%), strong staining was observed both in the inner epithelium of the enamel organ and in the adjacent stellate reticulum. 16.7% of the AM cases showed MOC-31 expression, the immunoexpression expression was diffuse at the cytoplasmic and membrane level. The only two cases of ameloblastic carcinoma included were strong positive to MOC-31. No correlation was observed between protein expression and gender, age, clinical variants, or histological subtypes.

**Conclusions:**

Overexpression was found in TG and ameloblastic carcinoma compared to AM; further studies with different experimental strategies are suggested to clarify the biological significance of this finding.

** Key words:**Tooth germ, ameloblastoma, Ep-CAM, MOC-31, immunohistochemistry.

## Introduction

Ameloblastoma (AM) is an epithelial, benign odontogenic neoplasia, slow growing and progressive, which derives from the odontogenic epithelium, within a fibrous stroma (1). Its global incidence, taking into account studies from 1995 until 2018, and compared with a previous revision by Reichart *et al*. (1995), is estimated in 0,92 cases per million yearly (2). It is the most frequent odontogenic epithelial neoplasia in developing countries. In the western hemisphere, it is the second most frequent odontogenic tumor, after odontoma, while recent research in South America put it as the most frequent odontogenic tumor in this continent (3). Despite its slow growth, AM is characterized by local invasion and high recurrence rates. It is also characterized by causing facial deformity and asymmetry, leading to severe clinical consequences, associated in particular with late diagnosis (4). In 2017, the WHO classification of head and neck tumors described the following AM variants: conventional, unicystic, extraosseous / peripheral and metastasizing (5).

MOC-31 Clone recognizes the protein called Epithelial Cell adhesion molecule (Ep-CAM), also known as Adenocarcinoma-associated antigen, Cell surface glycoprotein Trop-1, Epithelial glycoprotein 314, KS 1/4 antigen, Major gastrointestinal tumor-associated protein GA733-2 and Tumor-associated calcium signal transducer 1. Ep-CAM is a 40 kDa transmembrane glycoprotein, with a 289 aminoacids extracellular domain, EpEX, and a short, 26 AA intracellular domain, EpICD (6). It is expressed in the basolateral membrane of most normal epithelial tissues, except in adult squamous epithelium and some specialized epithelial cell types such as hepatocytes (7), and it becomes overexpressed in tumors of epithelial origin (8). Ep-CAM plays an important role in diverse processes related to the development, maintenance, repair and function of epithelial tissues in the organism, among them: adhesion, proliferation, differentiation and cell cycle regulation, migration and cell signaling (9). The presence of this protein in neoplastic tissues seems to have different biological implications depending on the type of neoplasia studied (10). Ep-CAM is overexpressed frequently in a majority of malignant neoplasia (11). Several studies have identified the overexpression of Ep-CAM as related to processes of initiation, tumor progression, metastasis and poor outcome in pancreatic, gallbladder, gastric and nasopharyngeal cancer (11,12). In other cancers, however, Ep-CAM overexpression is associated with a better outcome, such as renal cell, thyroid or head and neck squamous cell carcinomas (13). However, it has been revealed that EpCAM could be a tumor suppressive protein in certain types of cancers (14,15). Hwang *et al*., suggest that decreased expression of EpCAM is an early event in oral carcinogenesis (10). The molecular mechanisms of the tumor suppressive function of EpCAM in these cancers are not yet clear.

Based on the existing literature, it would be expected that there would also be expression of Ep-CAM in odontogenic tumors of epithelial origin.

In this study, we have evaluated for the first time the expression of Ep-CAM (clone MOC-31) in tooth germ (TG), AM and ameloblastic carcinoma. We evaluate the presence of Ep-Cam in the normal embryological tissue (TG) and compare the results with the neoplastic tissue (AM) and ameloblastic carcinoma, aimed to suggest a possible function of Ep-Cam in the odontogenic and neoplastic process.

## Material and Methods

**Figure 1:**
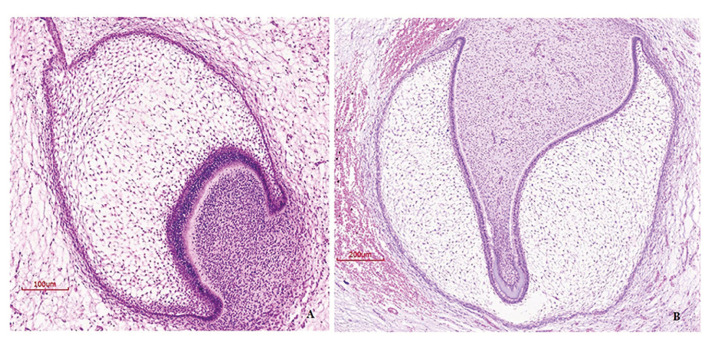
Hematoxylin-eosin staining for tooth germ. A) Early cap stage, magnification 200X B) Bell stage, magnification 100X.

16 paraffin embedded samples of human TG (from 12 different individuals) were selected in this study (stages of bud, cap and bell) (Fig. 1), obtained from the archives stored at the Histology department of the School of Dentistry, Universidad de la República (Uruguay), 60 tumor samples embedded in paraffin from AM and two ameloblastic carcinomas, diagnosed until 2014, from Latin American Oral Pathology services from Mexico (Universidad Juarez del Estado de Durango), Chile (Universidad de Chile).

All samples were re-assessed and classified by two experienced pathologists, in agreement with the criteria from the most recent WHO classification, of 2017 (5).

- Immunohistochemistry

For immunohistochemical analysis, 2 μm sections were heat-retrieved with 10 mM sodium citrate solution (pH 6.2) to expose antigenic epitopes. Endogenous peroxidases were quenched with 0.9% H2O2 for 5 min. Sections were incubated 45 min with MOC-31 primary antibody (Dako, Monoclonal Mouse Anti- Human Epithelial Related Antigen MOC- 31, 1:100 dilution). After primary antibody incubation, sections were incubated with a biotinylated secondary antibody followed by a streptavidin-horseradish peroxidase complex (LSA-B + Labeled streptavidin-biotin, Dako Corporation, Carpinteria CA, USA), 30 min each. The reaction products were visualized using the 3,3’-diaminobenzidine-H2O2 substrate (Biocare Medical), and the sections were counterstained with Mayer’s hematoxylin. Kidney normal tissue and human breast adenocarcinoma were used for the positive control. For the negative control sections, the primary antibody was omitted.

Sections were visualized in their totality with an optical microscope (Eclipse CI-L, Nikon, Japan), and positivity observed in cell membrane and cytoplasm was graded according to the following semi-quantitative scale: Neg, "essentially unstained", for negative sections or staining <5% of cells; + for staining of 5-50% of cells; ++ for staining >50% of cells (16).

As clone MOC 31 had never been tested in ameloblastic tissue, it was decided to perform a second confirmatory technique in 2 cases.

Western Blot

In two cases of AM, protein extraction was performed using a Qproteome FFPE tissue kit (Catalog Number 37623, QIAGEN, Hilden, Germany), following the manufacturer’s recommendations. The protein concentration was determined using the Bradford method with a spectrophotometer DeNovix DS-11. From each extraction, only 10 to 20 μg of protein were used and separated on 12% PAGE/SDS (polyacrylamide gel electrophoresis/sodium dodecyl sulfate), 100 V for 30 minutes. Electrotransference to polyvinylidene difluoride membranes over 2 hours at ambient temperature was performed (Hoefer Blot Module). Blockage was performed using buffer TNE (10 mM Tris-HCl, pH 7.5; 2.5 mM EDTA, pH 8.0, and 50 mM NaCl), 1% Tween-20, and ambient temperature for 1 hour. Three washes were performed for 5 minutes each with TBST buffer. Incubation of the primary antibodies anti-MOC 31 (1:1100), and anti-α-actin (1:500) with the membrane was performed at 4°C for 2 hours under soft stirring. Then, the secondary antibody (goat-anti-mouse-HRP conjugated secondary antibody) was added at room temperature (diluted 1/5000) for 1 hour under soft stirring. Detection was performed using an Opti-4CN substrate kit (Catalog Number 970-3210, BIORAD, Hercules, CA).

The results were analyzed descriptively. The presence/absence of association between MOC-31 staining and gender or age was determined using Pearson's chi-squared test. The strength of association was determined by the contingency coefficient. The results were considered significant at *p* < 0.05. Statistical procedures were performed with R software.

## Results

Regarding clinical information with gender and age distribution, 58,3% of AM cases were female, whereas the mean age in this group was 39.1, (SD +18,8) years.

Distribution of AM cases using the 2017 WHO classification was (5): conventional 71.2%, unicystic 22% and peripheral 6.8%. Regarding histological subtypes, the following distribution was observed: in conventional AM, 36.7% cases were plexiform, 25% were follicular, 6.7% were acanthomatous and 1.7% were desmoplastic. In unicystic AM, 11.7% cases were luminal, 1.7% were intraluminal and 8.3% were mural. The rest of the case were peripheral (6.7%).

**Figure 2:**
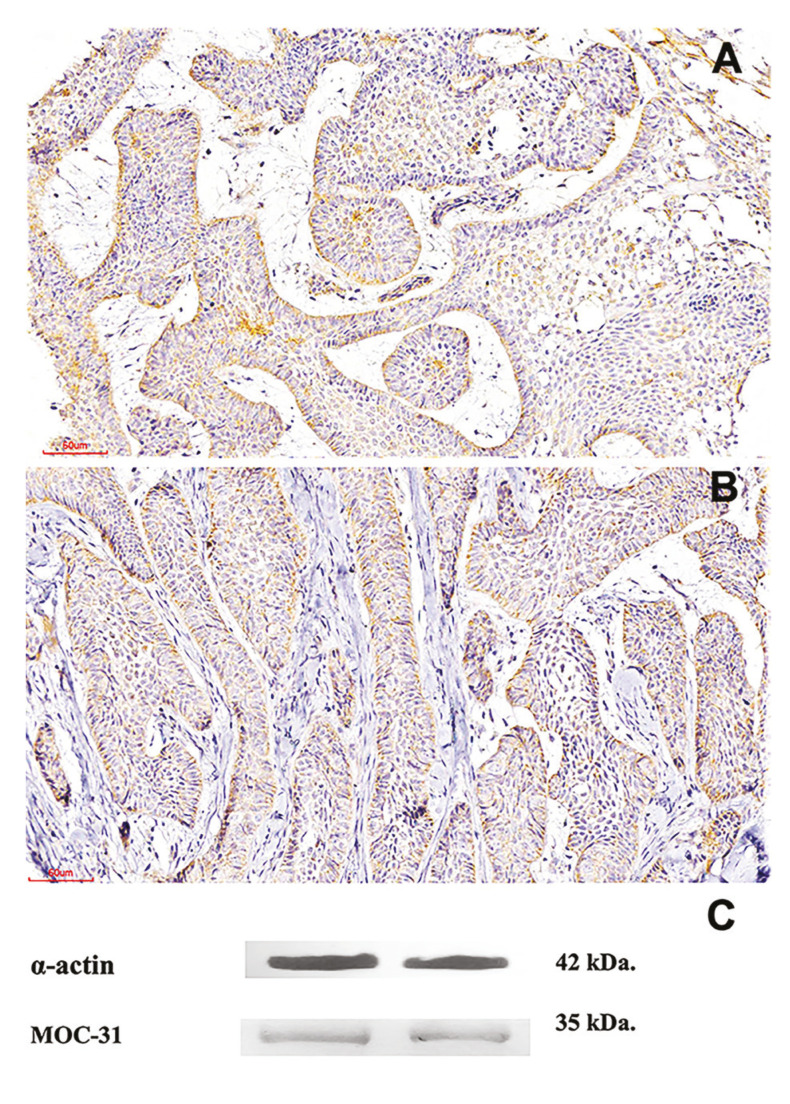
MOC-31 expression in ameloblastoma. A-Strong staining in epithelial islets. B-Moderate staining at the epithelial level. 200X. C- Western blot detection of MOC-31 clone and the positive control α-actin in ameloblastomas.

**Figure 3:**
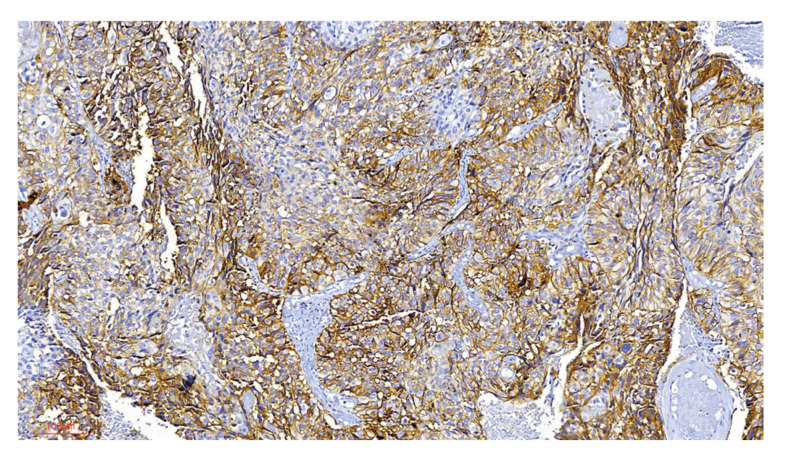
MOC-31 expression in ameloblastic carcinoma. A-Strong staining in malignant epithelial cells. 200X.

Some of the samples used in this research were incisional biopsies, and the final diagnosis after surgical treatment was not evaluated. Therefore, the final classification of unicystic ameloblastoma maybe not exact, which is limitation of our study. Cytoplasmic and membrane expression of MOC-31 in AM with a staining score of ++ was observed in 4 (6.7%) cases, with a score of + in 6 (10%) cases and was negative in 50 (83.3%) cases. MOC-31 expression was diffuse at the cytoplasmic and membrane level (Fig. 2, Fig. 3). Immunostaining comparation for MOC-31 in TG and AM is summarized in Table 1.

MOC-31 expression regarding gender, age, clinical variant, and histological subtype within AM is described in Table 2. No statistically significant association was found between MOC-31 expression and gender, age, clinical variant, or histological subtype in the AM group, as shown in particular by the low values of the CC.

**Figure 4:**
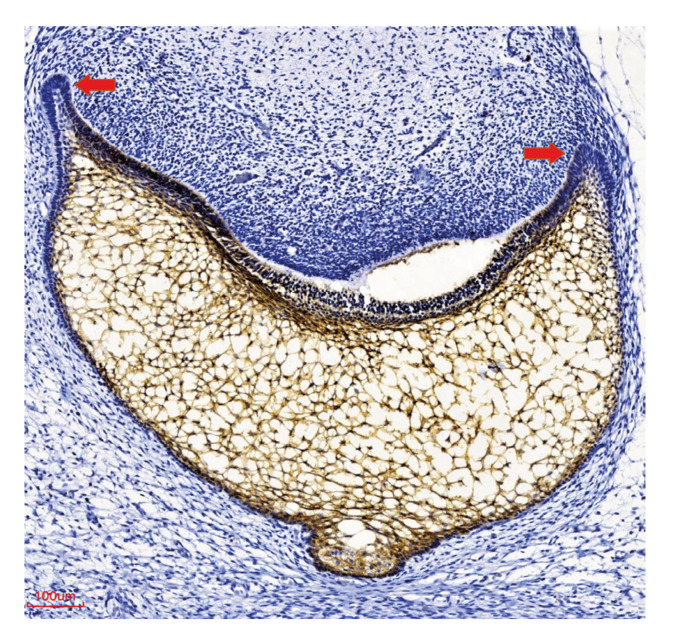
Expression of MOC-31 in tooth germs in the cap stage. Diminished expression is observed in the cervical loop (arrows). Dental papilla is negative. 200X.

Of the 16 TG cases analyzed, nine (56,3%) had a MOC-31 staining score of ++, four (25%) had a score of +, and three (18.8%) were negative (Table 1, Table 3). Strong staining was observed both in the inner epithelium of the enamel organ and in the adjacent stellate reticulum (Fig. 4). The two cases of ameloblastic carcinoma included in this study were strong positive (++), (Fig. 3).

In the TG group, the distribution of cases according to developmental stage was as follows: bud, 18.7%; cap, 43.7%; and bell, 37.8%. Table 3 describes MOC-31 expression according to developmental stage in the TG group (Table 3).

**Table 1:**
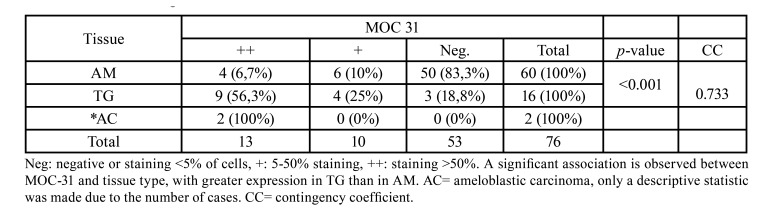
MOC-31 staining scores.

**Table 2:**
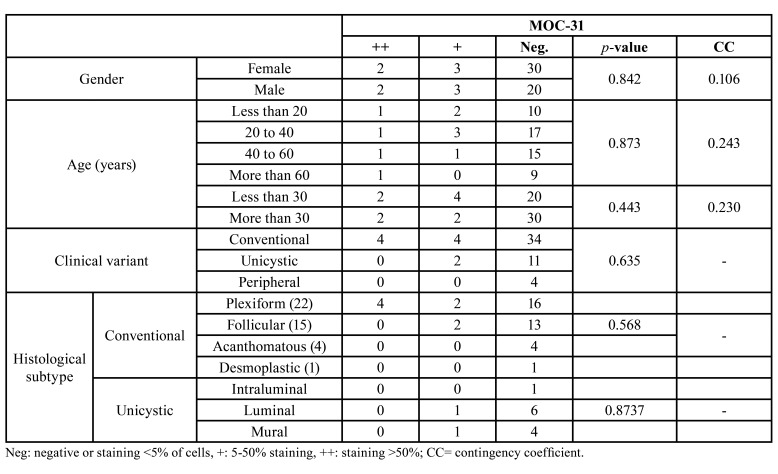
MOC-31 expression in relation to gender, age, clinical variant, and histological subtype in AM.

**Table 3:**
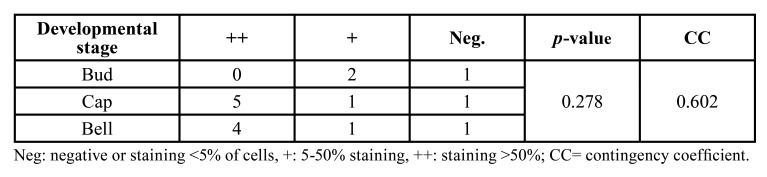
MOC-31 expression according to developmental stage in the TG group.

Regarding an eventual relationship between MOC-31 expression and developmental stage in TG, we did not observe a statistically significant association between these variables, even though a moderate value of CC was obtained.

As clone MOC 31 had never been tested in ameloblastic tissue, it was decided to perform a second confirmatory technique in 2 cases. Western blot analysis of MOC-31 (35 kDa) protein expression and α-actin (positive control) was positive in the samples included (Fig. 2).

## Discussion

Tissue and organ morphogenesis occurs as a result of interactions between different cell populations. An important type of intercellular interaction fundamental for tissue morphogenesis is cell adhesion, mediated by cell adhesion molecules which also play important roles in a variety of dynamic processes, such as cell migration, proliferation, and differentiation (17). Cell adhesion systems are therefore key participants in the processes that organize tissue structure and development (17).

Even though Ep-CAM expression is normally expressed in epithelial tissues (6,7), it is also present in stem cells during development, a characteristic shared with other cell adhesion molecules (18), which are generally expressed from the first stages of development in a tissue specific manner (19). Ep-CAM typically is not expressed in terminally differentiated, adult epithelial cell types, such as keratinocytes and pancreatic islets (20).

To our knowledge, this is the first study that compares the expression of Ep-CAM between normal embryonic tissue of odontogenesis (TG) and neoplastic odontogenic tissue (AM and ameloblastic carcinoma).

In our study, Ep-CAM expression in tooth germ was positive in 81.3% of cases, in agreement with studies that show its expression in other epithelia during different organs morphogenesis (20). Odontogenesis is an embryological process where the tissue is in constant growth and structural modification. In this study we found strong expression of Ep-CAM in the inner enamel epithelium and in the stellate reticulum which could be related to the importance of cell cohesion in different developmental events that occur in these regions. However, with the methodology used in this study we can only limit ourselves to describing the presence of this adhesion molecule during the odontogenesis process. Studies with other experimental approaches using genetic animal models are needed to be able to define exactly what the Ep-CAM functions are in odontogenesis. and identifying the underlying molecular mechanisms.

The expression of cell adhesion molecules is altered in several neoplasia, particularly in malignant ones, favoring progression, invasion, recurrence, and metastasis (21).

During and after malignant transformation, aberrant expression, such as downregulation, overexpression, or de novo expression, has been observed for different cell adhesion molecules (21). Ep-CAM is overexpressed in a variety of carcinomas, including the metastatic stage (11), possibly promoting sustained proliferation, tumor growth and metastasis (12). Ep-CAM expression has been observed in tumor initiating cells and in disseminated tumor cells, making this protein a candidate target for tumor diagnosis and therapy (22). In most malignant neoplasms of epithelial origin, over-expression of Ep-CAM has been found and this is associated with a poor prognosis, however in the case of gastric cancer, some studies also suggest that Ep-CAM overexpression is associated with a better prognosis, and a consensus on the role of this protein has not been attained (23,24).

Ep-CAM roles in the progression of different types of tumors could be linked to the specific biological characteristics of each tumor (25), and there are still many aspects concerning its involvement in tumor development and prognosis that remain to be elucidated (26).

AM is the most frequent odontogenic, benign neoplasia of epithelial origin, and its locally aggressive, invasive, and highly recurrent behavior represents an important challenge towards its clinical management (3).

Our study showed negative expression of Ep-CAM in 83.3% of cases of AM. No correlation was observed between Ep-CAM expression and the different clinical variants or histological subtypes of the AM cases analyzed, nor in relation to gender and age of patients.

Based on the literature, we would have expected higher expression of Ep-CAM in AM, however we found poor or no expression of this protein in the neoplastic tissue.

It should be noted that despite being locally aggressive and locally destructive, AM continues to be a benign neoplasm. Finding this unexpected result, we decided to include in the study also the malignant counterpart of AM; ameloblastic carcinoma, finding strong positivity in the only two cases included, this over-regulation of Ep-CAM seems to coincide with what happens in most of the epithelial malignant neoplasms. However, we must be cautious since such a small sample does not allow us to draw any conclusions.

As a future strategy, it would be useful to study and compare the expression of Ep-CAM in a larger casuistic of malignant epithelial odontogenic neoplasms such as ameloblastic carcinoma.

## Conclusions

Ep-CAM is highly expressed in tooth germ, whereas it is markedly downregulated or negative in AM and highly expressed in ameloblastic carcinoma. We consider that knowledge of Ep-CAM expression in odontogenic neoplasia, such as AM and ameloblastic carcinoma can provide valuable information towards a better understanding of the biology of these neoplasias. Further studies with different experimental strategies will be necessary to precisely define the roles of Ep-CAM downregulation in the development and progression of AM.
